# Chemical Application of Topological Indices in Infertility Treatment Drugs and QSPR Analysis

**DOI:** 10.1155/2023/6928167

**Published:** 2023-11-24

**Authors:** Sobia Sultana

**Affiliations:** Department of Mathematics and Statistics, Faculty of Science, Imam Mohammad Ibn Saud Islamic University (IMSIU), P.O. Box 90950, Riyadh 11623, Saudi Arabia

## Abstract

The main challenges faced by medical researchers while producing novel drugs are time commitment, amplified costs, creating a safety profile for the medications, reduced solubility, and a lack of experimental data. Chemical graph theory makes an important theoretical contribution to drug development and design by investigating the structural properties of molecules. To improve drug research and assess the effectiveness of treatments, topological indices aim to provide a mathematical representation of molecular structures. In this study, the author examined a number of recently used drugs, including tamoxifen, mesterolone, anastrozole, and letrozole which are used to treat infertility. We compute the topological descriptors with the limiting behaviors associated with these pharmaceutical drugs and offer degree-based topological parameters for them. We conducted a QSPR investigation on the prospective degree-based topological descriptors using quadratic, cubic, exponential, and logarithmic regression models.

## 1. Introduction

**Figure 1:**
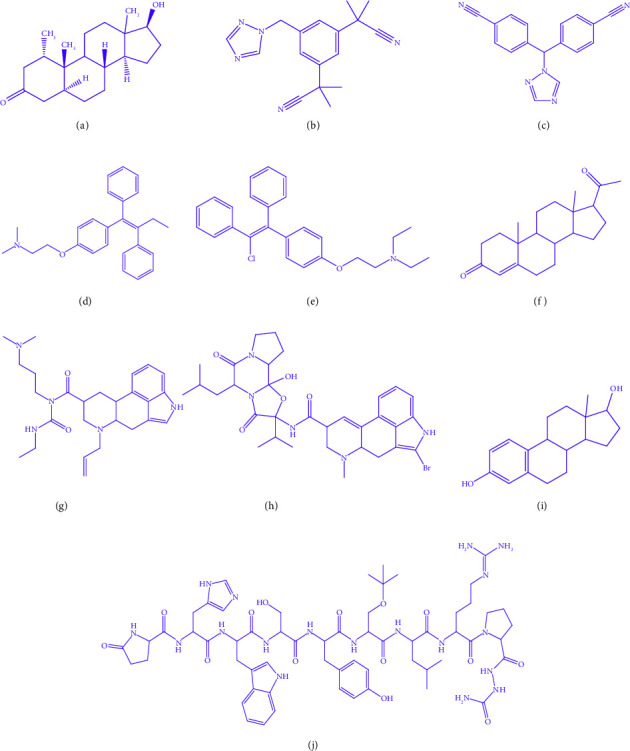
Molecular structure. (a) Mesterolone. (b) Anastrozole. (c) Letrozole. (d) Tamoxifen. (e) Clomifene. (f) Progesterone. (g) Cabergoline. (h) Bromocriptine. (i) Estradiol. (j) Goserelin.

According to consensus among the majority of specialists, infertility is defined as the inability to conceive after actively trying for at least a year. Infertility is a reproductive system disorder that prevents an individual from getting pregnant. Children play a key role in enabling their parents to contribute to the survival of the family, the culture, and the community. Most communities, especially in emerging nations, are designed with the expectation that younger generations will care for and support older family members in the future [1]. The availability of infertility cures is a component of Millennium Development Goal number 5, and infertility has been acknowledged by the WHO as a public health issue [2]. Worldwide, approximately eighty million people suffer from impaired fertility, with prevalence rates ranging from less than five percent to over thirty percent. By 2025, 7.7 million more people are anticipated to experience infertility, despite advancements in reproductive technologies [3]. Male infertility is most frequently caused by abnormalities in the sperm shape, motility, or quantity (low sperm count) of sperm. The majority of cases of infertility in people with ovaries are brought on by ovulation abnormalities, where ovulation is the process during which ovary releases an egg, getting it ready for potential fertilization by sperm. Approximately, 10–15% of couples in the reproductive age group face infertility, which differs from other health-related conditions as it also involves psychological and social factors. Approximately, 40–45% of cases of infertility are caused by men, either alone or in conjunction with their female spouses [4]. The rising interest in reproductive treatments has led to increased awareness and motivation for research regarding the psychological effects of infertility. The connection between mental illness and infertility has been taken into account. In addition, studies have examined the psychological impacts of infertility itself along with chronic exposure to intrusive infertility therapies on mental health. Developing new pharmaceuticals is costly, time-consuming, and perplexing in this area. In addition, it requires quick diagnosis and treatment to address the condition. Among the ten selected medications that are essential for the community's health, safe, and highly effective, the medications include mesterolone, anastrozole, letrozole, tamoxifen, clomifene, progesterone, cabergoline, bromocriptine, goserelin, and estradiol.  Figure 1 depicts the aforementioned medications.

TIs (topological indices) are distinct numerical descriptors derived from graphs that represent a chemical structure perfectly. These are effectively used to describe the physical characteristics of several medications. To achieve this, a variety of TIs and polynomials are used, and they accurately represent the veiled facts in graph theory. Every TI is significant and exhibits a notable function in chemical graphs. The use of graph invariants (TIs) in investigations of quantitative structure-property relationships (QSPR) and quantitative structure-activity relationships (QSAR) has attracted significant attention in recent years. Topological indices are employed frequently in various domains such as mathematics, bioinformatics, and cheminformatics, but their most extensive utilization is observed in the field of QSPR. The optimal association between TIs and pharmacological characteristics can be determined using QSPR models.

The author has determined degree-based TIs for infertility medicines in the current research paper. Similarly, topological indices and imposed QSPR (models) are used for comprehensive analysis. These TIs and drug characteristics have been estimated using a linear regression model (LRM). The estimation of certain of these medications' physicochemical properties, or TIs, is also employed in the creation of QSPR models. Curve fitting was used in the QSPR research, which found a strong connection between the features of anticancer drugs. The comprehensive examination of skin cancer drugs is conducted using Khan and Nadeem's imposed QSPR modeling. They have demonstrated the importance of topological indices in understanding the physical characteristics of medications [5]. To anticipate the physicochemical characteristics of medications to treat diabetes, Parveen et al. [6] used the QSPR model. Earlier research on potential drugs for the treatment of COVID-19 was covered by Colakoglu. Given that discovery is a costly and complex phenomenon, this technique works best for predicting it [7]. Targeted analysis and painstakingly crafted topological indexes were used by Parveen et al. to evaluate RA medications [8]. Nasir et al. demonstrate a strong association between the properties of the pharmaceuticals and TIs in their results on blood cancer treatments using QSPR modeling [9]. Frequent studies have initiated a direct association between the chemical properties and molecular structures of chemical compounds and pharmaceuticals. Topological indices are used for detailed investigations in the modeling of cardiovascular medicines [10]. Drugs for vitiligo caused by autoimmune illness were discussed in [11].

Zaman et al. [12] conducted a thorough investigation of the neighborhood version of the sudoku nanosheet topological indices. The issues with traditional drug delivery, such as poor solubility, toxicity, and irregular drug release, can be solved with the use of new technologies based on nanomaterials or naphtalenic nanosheets. Ullah et al. [13]. Degree-based TIs are used by Zaman et al. in [14] to establish a best-fit regression model using QSPR, and they came to the conclusion that the indices are useful in predicting the characteristics of blood cancer medications. For cerium oxide nanostructures, modified versions of the Second Zagreb index and other indices are computed by Zaman et al. [15]. One of the supramolecular dyes employed in Masson's trichrome stain, fuchsine acid, has a wide range of uses in histology. As an organic semiconductor, it has numerous other significant uses in electronic fields and photonic devices.

With the ultimate goal of shedding light on the efficacy of the computed molecular descriptors for QSAR and QSPR investigations carried out by Ullah et al. [16], closed equations are derived for some of its significant irregular molecular descriptors. Ullah et al.'s [17] investigation is made simple by the engendered formulas and mathematical verdicts that are obtained. A number of graphs with excellent application-graph perspectives were estimated by Zhou et al. in [18], paving the path for new and established results in this field. However, employing distance-base Eigen values and sign-less Laplacian energy of graphs, Indulal et al. in [19] produced significant findings. This was succinctly explained by Kirmani et al. in [20]. Generalization of descriptors may decrease the quantity of molecular graph-based descriptors while simultaneously improving current findings and offering a stronger link to multiple molecular features. COVID-19 is a worldwide issue that is being studied and treated with a number of medications. Zhong et al. explore the illness medications by imposing QSPR modes and using topological indices to aid in their investigation [21]. Jovanović and Stanić additionally take into account the spectral distances bounded by a certain constant in [22]. The aforementioned works have inspired us to explore the current study of infertility drugs using topological indices.

## 2. Methods

The molecular graph depicts a molecular structure made up of a set of atoms or vertices *V*(*G*), which are joined by a set of bonds or edges *E*(*G*). In a molecular graph, size of graph is *n* and order *m* refers to the total number of atoms, or vertices, and the total number of bonds, or edges, respectively. Graph theory and chemistry are often employed to address various chemical graph problems. Topological indices play a crucial role in QSPR analysis as well as in the domains of chemical graph theory and mathematical chemistry. The TIs we used were as follows:

**Definition 1 .**

ABC index [23] and Randic index [24] of *G* are given under

(1) $$\begin{array}{c} \text{ABC}\left(G\right)=\underset{uv\in E\left(G\right)}{\sum }\sqrt{\frac{d_{u+}d_{v}-2}{d_{u}d_{v}}},\\ \text{RA}\left(G\right)=\underset{uv\in E\left(G\right)}{\sum }\sqrt{\frac{1}{d_{u}d_{v}}}. \end{array}$$

**Definition 2 .**

Sum connectivity index [25] and GA index [26] of *G* are given under

(2) $$\begin{array}{c} S\left(G\right)=\underset{uv\in E\left(G\right)}{\sum }\sqrt{\frac{1}{d_{u}+d_{v}}},\\ \text{GA}\left(G\right)=\underset{uv\in E\left(G\right)}{\sum }\frac{2\sqrt{d_{u}d_{v}}}{d_{u}+d_{v}}. \end{array}$$

**Definition 3 .**

Harmonic index [27] and hyper Zagreb index [28] of *G* are given under

(3) $$\begin{array}{c} H\left(G\right)=\underset{uv\in E\left(G\right)}{\sum }\frac{2}{d_{u}+d_{v}},\\ \text{HM}\left(G\right)=\underset{uv\in E\left(G\right)}{\sum }{\left(d_{u}+d_{v}\right)}^{2}. \end{array}$$

**Definition 4 .**

Forgotten index [29] is defined as follows:

(4) $$\begin{array}{c} F\left(G\right)=\underset{uv\in E\left(G\right)}{\sum }\left[{\left(d_{u}\right)}^{2}+{\left(d_{v}\right)}^{2}\right]. \end{array}$$

Mesterolone with a chemical formula C_20_H_32_O_2_ is a steroid used for the treatment of low testosterone levels, and it exerts minimal impact on sperm counts and levels. Anastrozole, with the chemical formula C_17_H_19_N_5_, serves as a nonsteroidal inhibitor prescribed for adjuvant therapy in postmenopausal women. It is used to reduce circulating estrogen. Aromatase inhibitors, including anastrozole, have become the preferred endocrine medications for postmenopausal breast cancer treatment. Letrozole is prescribed for the treatment of postmenopausal women, with a chemical formula of C_17_H_11_N_5_. Tamoxifen, with the chemical formula C_26_H_29_NO is a selective estrogen receptor modulator used in these treatments either alone or as an adjuvant. Clomifene is a medication used to stimulate ovulation, and its chemical formula is C_26_H_28_ClNO. Clomifene (formerly clomifene) is an ovulatory stimulant that is taken orally and operates as a selective estrogen receptor modulator. Clomifene can cause multiple ovulations, thereby increasing the likelihood of having twins. There is a potential for an elevated risk of ovarian cancer and weight gain. Clomifene can interact with tissues that have estrogen receptors such as the hypothalamus, pituitary, ovary, endometrial, vagina, and cervix. Cabergoline is used to treat hyperprolactinemic disorders caused by various factors, and its chemical formula is C_32_H_40_BrN_5_O_5_. Goserelin is used to treat breast and prostate cancer by lowering pituitary gonadotropin output. Estradiol is used to treat vaginal atrophy in menopause breast cancer treatment and advanced androgen-dependent prostate cancer. The author has applied TIs on drugs in this article and obtained the required results. All the formulas for infertility dugs can be found at Pubchem and Chemspider.

## 3. Topological Indices Calculation

The author computes topological indices for the anastrozole (AZ) and partition of DU with edge set *E*. *E*_*m*,*n*_ are edges of AZ with |*E*_1,4_|=6, |*E*_2,2_|=3, |*E*_2,3_|=10, |*E*_3,4_|=2. By applying Definitions 1 to 4 we obtain results as follows:

- ABC(AZ) = $6\sqrt{\left(1+4-2\right)/\left(1\times 4\right)}+3\sqrt{\left(2+2-2\right)/\left(2\times 2\right)}+10\sqrt{\left(2+3-2\right)/\left(2\times 3\right)}+2\sqrt{\left(3+4-2\right)/\left(3\times 4\right)}=$ 15.68.
- $\text{RA}\left(\text{AZ}\right)=6\sqrt{1/\left(1\times 4\right)}+3\sqrt{1/\left(2\times 2\right)}+10\sqrt{1/\left(2\times 3\right)}+2\sqrt{1/\left(3\times 4\right)}=$ 9.16.
- $S\left(\text{AZ}\right)=6\sqrt{1/\left(1+4\right)}+3\sqrt{1/\left(2+2\right)}+10\sqrt{1/\left(2+3\right)}+2\sqrt{1/\left(3+4\right)}=9.41$.
- $S\left(\text{AZ}\right)=6\sqrt{1/\left(1+4\right)}+3\sqrt{1/\left(2+2\right)}+10\sqrt{1/\left(2+3\right)}+2\sqrt{1/\left(3+4\right)}=9.41$.
- $\text{GA}\left(\text{AZ}\right)=\left(12\sqrt{1\times 4}\right)/\left(1+4\right)+\left(6\sqrt{2\times 2}\right)/\left(2+2\right)+\left(20\sqrt{2\times 3}\right)/\left(2+3\right)+\left(4\sqrt{3\times 4}\right)/\left(3+4\right)=19.58.$
- *H*(AZ)=6(1/(1+4))+3(1/(2+2))+10(1/(2+3))+2(1/(3+4))=8.47.
- *HM*(AZ)=6(1+4)^2^+3(4+4)^2^+10(2+3)^2^+2(3+4)^2^=546.
- *F*(AZ)=6(1+9)+1(4+4)+18(4+9)+3(9+16)=306.

All other infertility drugs calculations are done with same procedure and are given in Table 1.

## 4. Dataset and Quantitative Structure Analysis and Regression Models

This section explores the use of topological indices and regression models to investigate the relationship between computed topological indices and physicochemical parameters. The author has tabulated computations of TIs and physical characteristics of molecular structures in Table 1, respectively. Regression models can be constructed using these values. The dataset for the aforementioned molecular structures includes the physicochemical characteristics found on ChemSpider, and the curves are fitted using regression models. In light of this, the author has investigated exponential, logarithmic, cubic, and quadratic models.

The square of the correlation coefficient's (*R*^2^), the F-ratio test, and significance (sig) were taken into account in the regression model table. The most accurate regression model is the one with the highest *R*^2^ value. The topological index regression models for the specific physicochemical attribute have a few best predictors, which have been highlighted here. The regression model is the best option for this investigation. Instead of fitting straight lines, various regression models have been used. This process is referred to as a curvilinear regression analysis. The following equations for curve fitting were examined in this study:

- *Y*=*a*+*b*_1_*X*_*i*_+*b*_2_*X*_*i*_^2^ (quadratic equation)
- *Y*=*a*+*b*_1_*X*_*i*_+*b*_2_*X*_*i*_^2^+*b*_3_*X*_*i*_^3^ (cubic equation)
- *Y*=*a*+*b*. ln*X*_*i*_ (logarithmic equation)
- *Y*=*a*.*b*^*X*_*i*_^ (exponential equation)

where *Y* is dependent variable, *a* is the regression model constant, **X**_**i**_(*i*=1,  2,  3,…) are independent variables, and **b**_**i**_(*i*=1,  2,  3,…) are the coefficients for descriptor. The SPSS and MATLAB are useful for determining the results.

**Figure 2:**
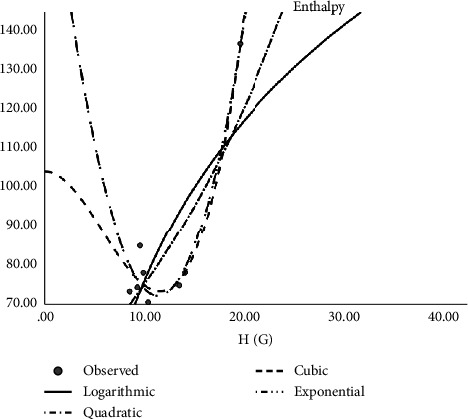
Logarithmic regression model of H(G) with enthalpy.

**Figure 3:**
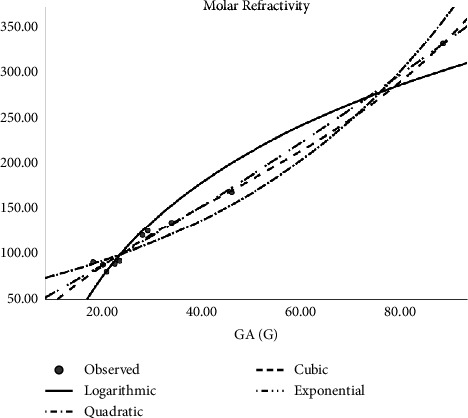
Logarithmic regression model of GA(G) with refractivity.

**Figure 4:**
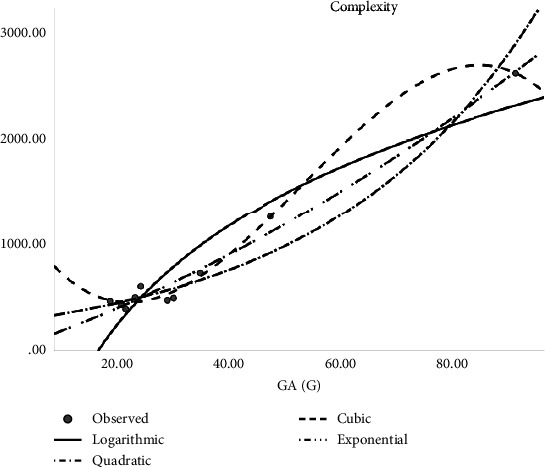
Logarithmic regression model of GA(G) with complexity.

**Figure 5:**
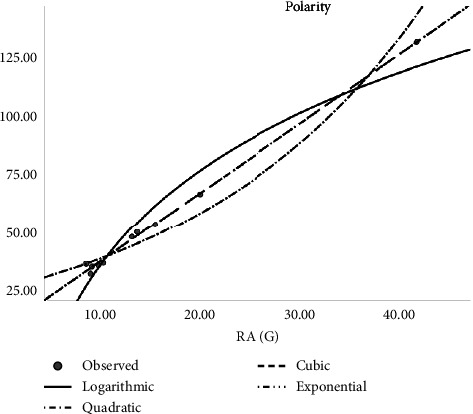
Logarithmic regression model of RA(G) with polarity.

**Figure 6:**
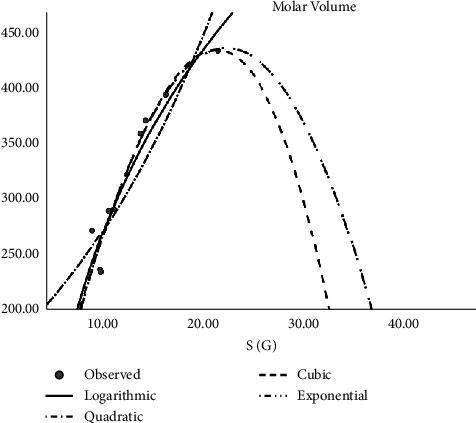
Logarithmic regression model of S(G) with molar volume.

In Table 2, the regression models for the enthalpy of molecular structures are presented whereas Figure 2 presents a logarithmic regression model portraying the association between H(G) and enthalpy, and Table 3 displays the regression models developed for predicting the boiling points of various molecular structures. The table includes coefficients, statistical metrics, and other relevant information essential for understanding the relationships between molecular features and boiling points. The models for predicting molar refractivity of molecular structures are summarized in Table 4. The table provides a comprehensive overview of the regression coefficients, their significance, and any additional parameters employed in the models.  Figure 3 illustrates the logarithmic regression model depicting the relationship between GA(G) and refractivity. As can be seen in Table 5, the author has constructed regression models that combined the aforementioned topological indices with the physical characteristics of molecular structures. Here Table 5 outlines the regression analysis for assessing the complexity of molecular structures. The logarithmic regression model in Figure 4 demonstrates the relationship between GA(G) and molecular complexity. The regression models for predicting the polarity of molecular structures are detailed in Table 6 while Figure 5 provides a visual representation of the logarithmic regression model depicting the relationship between RA(G) and polarity. In Figure 6, the logarithmic regression model illustrates the relationship between S(G) and molar volume.

## 5. Conclusions

The author examined correlation coefficients between the topological indices and other physical characteristics of drugs used to treat infertility, which demonstrate how well the aforementioned indices serve as predictors. In particular, the study presents a quantitative structure-property relationship (QSPR) analysis of molecular descriptors (TIs), which are tools used to predict the physical and chemical characteristics of drugs, especially in the context of pharmaceutical and medical applications. It is worth noting that molar refractivity and complexity are reliable indicators for these predictions. However, the estimation of polarity and polar surface area is less dependable.

- In a quadratic and cubic regression model, the molecular descriptor S(G) is most accurately predicted by factors such as polarity, molar volume, and complexity.
- Molecular descriptor ABC(G) is best predicted by refractivity when using a logarithmic regression model.
- On employing an exponential regression model, molar refractivity is the most effective predictor for the molecular descriptors GA(G) and HM(G).

Summing up, the topological indices and the structural properties of infertility medication compounds have great and strong connections. The correlation coefficients observed in QSPR modeling are located in close proximity to 1. The observed result shows that *p* value is below 0.05 and F-test value exceeds 2.5. These conditions confirm the validity of authors' findings. Both experimental and theoretical model results are highly consistent with one another. The author evaluates the predictive power of the degree base TIs using the physicochemical features of these structures. Our understanding of chemistry, pharmacy science, and drug discovery will all be improved by the findings of this study. Employing the study's findings, information on a certain chemical molecule can be found without the need for experiments if it is synthesized from these.

This research sets the stage for future investigations to calculate TIs for newly developed medications, providing valuable insights into their chemical structures and properties. Such knowledge can be crucial for the development and optimization of pharmaceutical compounds. This also enhances the field of topological analysis and provides researchers with extra resources for studying molecule properties by introducing other indices.

In future, these indices can be utilized to many conversions of graphs and probe into other chemical networks and diseases drugs. The molecular structures can also be analyzed on the base of graph energies this will also yield valuable insight into drugs discovery in pharmaceutical industry [30].

## Figures and Tables

**Table 1:** Topological indices of infertility treatment drugs.

Drug	ABC	RA	S	GA	H	HM	F
Mesterolone	17.93	10.37	11.03	23.91	9.83	724	386
Anastrozole	15.68	9.16	9.41	19.58	8.47	546	306
Letrozole	15.65	9.74	10.20	21.53	9.50	502	260
Tamoxifen	21.27	13.69	14.13	29.45	13.40	652	336
Clomifene	21.87	14.22	14.65	30.51	13.97	670	344
Progestrone	18.66	10.86	11.53	24.88	10.31	738	394
Cabergoline	25.49	15.96	16.60	35.11	15.47	870	450
Bromocriptine	35.02	20.37	21.70	47.08	19.38	1380	728
Goserelin	67.42	41.79	42.93	89.33	39.87	2168	1152
Estrdiol	16.36	9.59	10.27	22.27	9.24	624	328

**Table 2:** Regression models for enthalpy of molecular structures.

Enthalpy				
Regression model	Molecular descriptor	*R* ^2^	*F*	Sig
Quadratic model	ABC(G)	0.968	75.655	0.000
	RA(G)	0.960	60.135	0.000
	S(G)	0.959	58.803	0.000
	GA(G)	0.959	58.458	0.000
	H(G)	0.950	47.272	0.000
	HM(G)	0.974	94.975	0.000
	F(G)	0.981	127.722	0.000

Cubic model	ABC(G)	0.967	73.368	0.000
	RA(G)	0.963	64.170	0.000
	S(G)	0.962	63.173	0.000
	GA(G)	0.962	62.761	0.000
	H(G)	0.958	56.399	0.000
	HM(G)	0.974	92.196	0.000
	F(G)	0.980	120.050	0.000

Logarithmic model	ABC(G)	0.710	14.666	0.009
	RA(G)	0.617	9.663	0.000
	S(G)	0.644	10.848	0.017
	GA(G)	0.669	12.116	0.013
	H(G)	0.583	8.394	0.027
	HM(G)	0.705	14.331	0.009
	F(G)	0.692	13.465	0.010

Exponential model	ABC(G)	0.779	21.127	0.004
	RA(G)	0.700	13.982	0.010
	S(G)	0.728	16.043	0.007
	GA(G)	0.754	18.415	0.005
	H(G)	0.670	12.161	0.013
	HM(G)	0.783	21.636	0.003
	F(G)	0.771	20.150	0.004

**Table 3:** Regression models for boiling point of molecular structures.

Molar volume				
Regression model	Molecular descriptor	*R* ^2^	*F*	Sig
Quadratic model	ABC(G)	0.951	58.831	0.000
	RA(G)	0.949	56.003	0.000
	S(G)	0.933	41.491	0.000
	GA(G)	0.910	30.174	0.001
	H(G)	0.926	37.681	0.000
	HM(G)	0.653	5.635	0.042
	F(G)	0.606	4.615	0.061

Cubic model	ABC(G)	0.952	58.864	0.000
	RA(G)	0.950	56.584	0.000
	S(G)	0.935	42.833	0.000
	GA(G)	0.913	31.611	0.001
	H(G)	0.928	38.591	0.000
	HM(G)	0.653	5.649	0.042
	F(G)	0.606	4.615	0.061

Logarithmic model	ABC(G)	0.895	59.577	0.000
	RA(G)	0.936	101.674	0.000
	S(G)	0.914	74.169	0.000
	GA(G)	0.886	54.228	0.000
	H(G)	0.921	81.308	0.000
	H(MG)	0.637	12.273	0.010
	F(G)	0.596	10.342	0.015

Exponential model	ABC(G)	0.768	23.209	0.002
	RA(G)	0.848	38.962	0.000
	S(G)	0.819	31.603	0.001
	GA(G)	0.784	25.464	0.001
	H(G)	0.851	40.100	0.000
	HM(G)	0.538	8.151	0.025
	F(G)	0.507	7.196	0.031

**Table 4:** Regression models for molar refractivity of molecular structures.

Molar refractivity				
Regression model	Molecular descriptor	*R* ^2^	*F*	Sig
Quadratic model	ABC(G)	0.991	143.296	0.000
	RA(G)	0.997	1202.58	0.000
	S(G)	0.995	701.649	0.000
	GA(G)	0.992	420.669	0.000
	H(G)	0.997	1052.56	0.000
	HM(G)	0.957	78.019	0.000
	F(G)	0.954	72.029	0.000

Cubic model	ABC(G)	0.991	368.122	0.000
	RA(G)	0.997	1202.59	0.000
	S(G)	0.995	703.808	0.000
	GA(G)	0.992	253.784	0.000
	H(G)	0.997	1052.56	0.000
	HM(G)	0.960	47.888	0.000
	F(G)	0.955	42.881	0.000

Logarithmic model	ABC(G)	0.947	143.2963	0.000
	RA(G)	0.948	146.356	0.000
	S(G)	0.942	130.513	0.000
	GA(G)	0.932	109.657	0.000
	H(G)	0.939	122.952	0.000
	HM(G)	0.840	42.080	0.000
	F(G)	0.835	40.352	0.000

Exponential model	ABC(G)	0.937	117.989	0.000
	RA(G)	0.947	141.900	0.000
	S(G)	0.947	142.275	0.000
	GA(G)	0.946	139.837	0.000
	H(G)	0.950	151.964	0.000
	HM(G)	0.886	62.339	0.000
	F(G)	0.875	56.014	0.000

**Table 5:** Regression for complexity of molecular structures.

Complexity				
Regression model	Molecular descriptor	*R* ^2^	*F*	Sig
Quadratic model	ABC(G)	0.985	226.123	0.000
	RA(G)	0.970	111.645	0.000
	S(G)	0.974	133.007	0.000
	GA(G)	0.979	166.938	0.000
	H(G)	0.964	94.310	0.000
	HM(G)	0.996	811.944	0.000
	F(G)	0.996	808.730	0.000

Cubic model	ABC(G)	0.985	226.123	0.000
	RA(G)	0.970	111.645	0.000
	S(G)	0.974	133.007	0.000
	GA(G)	0.992	245.379	0.000
	H(G)	0.964	94.310	0.000
	HM(G)	0.996	471.366	0.000
	F(G)	0.996	464.684	0.000

Logarithmic model	ABC(G)	0.919	91.217	0.000
	RA(G)	0.886	62.372	0.000
	S(G)	0.889	64.349	0.000
	GA(G)	0.890	64.922	0.000
	H(G)	0.867	52.338	0.000
	HM(G)	0.891	65.343	0.000
	F(G)	0.897	69.945	0.000

Exponential model	ABC(G)	0.924	97.467	0.000
	RA(G)	0.897	70.014	0.000
	S(G)	0.907	78.465	0.000
	GA(G)	0.919	91.290	0.000
	H(G)	0.889	64.375	0.000
	HM(G)	0.969	250.374	0.000
	F(G)	0.968	244.542	0.000

**Table 6:** Regression models for polarity of molecular structures.

Polarity				
Regression model	Molecular descriptor	*R* ^2^	*F*	Sig
Quadratic model	ABC(G)	0.991	373.563	0.000
	RA(G)	0.997	1230.037	0.000
	S(G)	0.995	717.807	0.000
	GA(G)	0.992	430.637	0.000
	H(G)	0.997	1082.986	0.000
	HM(G)	0.958	80.174	0.000
	F(G)	0.955	74.170	0.000

Cubic model	ABC(G)	0.991	377.832	0.000
	RA(G)	0.997	1230.45	0.000
	S(G)	0.995	7211.38	0.000
	GA(G)	0.992	261.142	0.000
	H(G)	0.997	1082.96	0.000
	HM(G)	0.961	49.523	0.000
	F(G)	0.957	44.359	0.000

Logarithmic model	ABC(G)	0.945	137.286	0.000
	RA(G)	0.946	139.512	0.000
	S(G)	0.940	124.906	0.000
	GA(G)	0.930	105.516	0.000
	H(G)	0.936	117.757	0.000
	HM(G)	0.838	41.479	0.000
	F(G)	0.833	39.869	0.000

Exponential model	ABC(G)	0.938	122.038	0.000
	RA(G)	0.949	147.538	0.000
	S(G)	0.949	147.707	0.000
	GA(G)	0.948	144.801	0.000
	H(G)	0.952	158.085	0.000
	HM(G)	0.888	63.354	0.000
	F(G)	0.877	56.941	0.000

## Data Availability

All the data are incorporated within the article, and there are no concealed or undisclosed datasets.
